# Blinding lights: Acute persistent vision loss in pregnancy

**DOI:** 10.1177/1753495X231200658

**Published:** 2023-09-24

**Authors:** Marie Leung, Ilia Ostrovski, Melin Peng-Franklin, Ahraaz Wyne

**Affiliations:** 1 Division of General Internal Medicine, 4257 Queen's University, Kingston, Canada; 2 Department of Medicine, 3710 McMaster University, Hamilton, Canada; 3 Department of Obstetrics and Gynecology, 3710 McMaster University, Hamilton, Canada

**Keywords:** Vision loss, retinopathy, milk-alkali syndrome, pancreatitis, pregnancy complications

## Abstract

Acute persistent vision loss in pregnancy is an emergent presentation with a broad differential and should prompt rapid assessment and treatment of the underlying etiology. In pregnancy, causes can include preeclampsia, severe gestational hypertension, and hemolysis, elevated liver enzymes, low platelets (HELLP) syndrome. Nonobstetrically related etiologies that can exacerbate in pregnancy include optic neuritis, giant cell arteritis, central retinal artery occlusion, or retinal detachment. In this case report, we describe a case of acute vision loss due to Purtscher's-like retinopathy, a rare but serious complication of pancreatitis in pregnancy. To our knowledge, this is the first published case of Purtscher's-like retinopathy in pregnancy unrelated to preeclampsia. Given the impact of permanent visual loss associated with Purtscher's-like retinopathy, more research is needed to determine treatments to substantively improve outcomes.

## Key Points

- Vision loss is a medical emergency with a broad differential (neurologic, ophthalmologic, systemic, or obstetrical-related etiologies) and should prompt rapid assessment.
- Purtscher's-like retinopathy is a rare but serious condition that can lead to permanent vision loss.
- Purtscher's or Purtscher's-like retinopathy occurs with systemic conditions such as trauma, pancreatitis, as well as obstetrical conditions such as severe gestational hypertension, preeclampsia, hemolysis, elevated liver enzymes, low platelets (HELLP) syndrome or post labor and delivery.

## Case summary

A 39-year-old multiparous Caucasian woman was pregnant for the third time and presented at 20 weeks and five days with nausea, vomiting, and abdominal pain.

She had a prior history of alcohol use disorder, abstinent for the past year, until she relapsed one week prior to admission. She had also been self-treating epigastric pain with high doses of over-the-counter antacids (calcium carbonate).

Past medical history was significant for gastroesophageal reflux disease, hypothyroidism, prior alcoholic hepatitis, osteopenia, and iron deficiency anemia.

Upon presentation, her vitals were: BP121/76 mmHg, HR 100 bpm, Temperature 36.4°C, SpO_2_ 93%. Physical examination was significant for epigastric tenderness and a palpable gravid uterus.

Investigations upon admission demonstrated: lipase 692 U/L (normal 10–140 U/L) and calcium level 4.55 mmol/L (ionized calcium 2.24 mmol/L), albumin 20 g/L, PTH 0.7 pmol/L (normal 2.0–9.4 pmol/L). Creatinine was 106 umol/L. Triglyceride levels were 1.96 mmol/L. Antinuclear antibody was negative. Blood and urine cultures were negative. She had no proteinuria. Abdominal ultrasound showed no evidence of gallstones, no intrahepatic or extrahepatic ductal dilatation and normal caliber common bile duct. The pancreas appeared normal with no inflammatory changes.

She was admitted for pancreatitis triggered by both alcohol and hypercalcemia. Her presentation demonstrated the triad of hypercalcemia, metabolic alkalosis and renal failure characteristic of milk-alkali syndrome, consistent with the reported use of up to 6000 mg/day of calcium carbonate.

Following admission, she reported worsening bilateral color distortions in her vision, describing the appearance of “tie-dye” in both her visual fields. Her visual acuity became limited, and she could only count fingers from a 2-foot distance in her right eye, and 1 foot distance in her left eye, at its worst. Her pancreatitis, renal failure, and hypercalcemia resolved over the ensuing days, however, her vision remained impaired.

**Figure 1.**
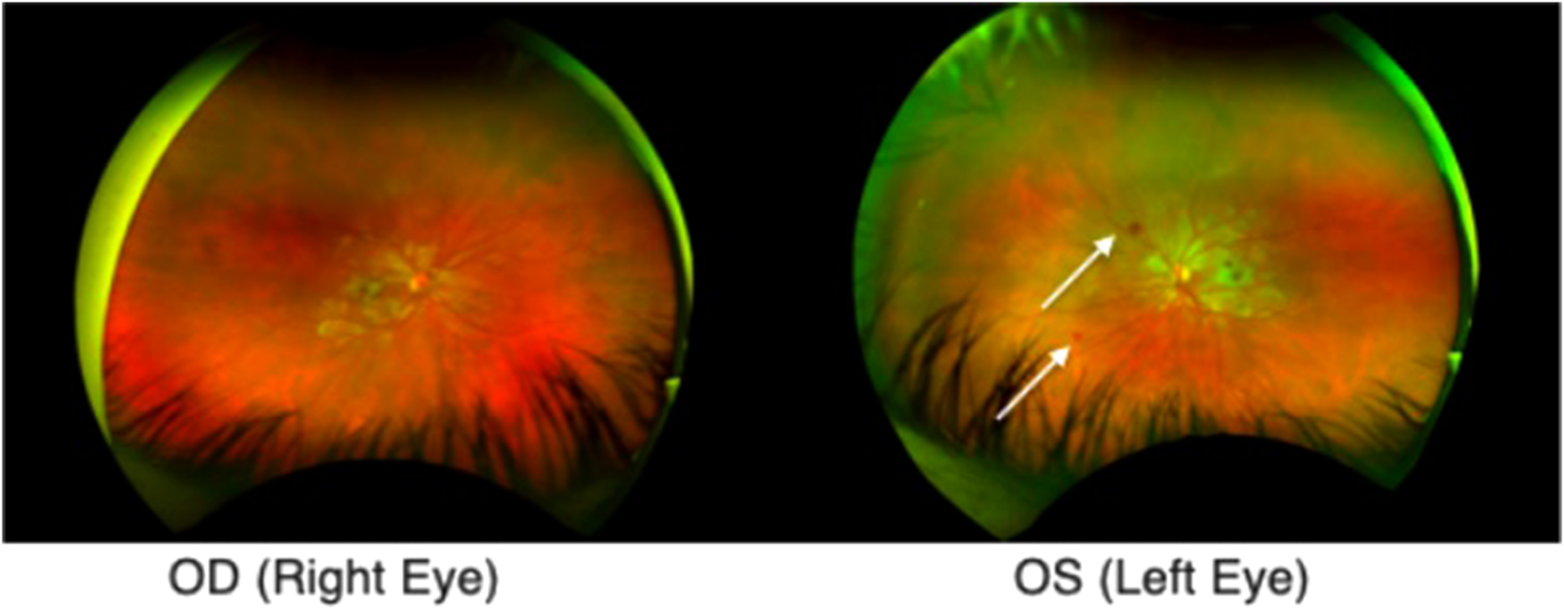
Ultrawide field images demonstrating bilateral whitening of Purtscher flecken with pseudo-cherry red spots and dot hemorrhages (white arrows).

CT scan of the head was unremarkable. Ophthalmologic consultation was sought and fundoscopy yielded extensive fleck-like retinal whitening and scattered retinal hemorrhages (Figure 1). She was diagnosed with Purtscher's-like retinopathy, a rare complication of pancreatitis.

Treatment was initially considered with systemic and local corticosteroids, but ultimately not pursued given minimal evidence for improving visual acuity or time to recovery. One month later, she had a partial resolution on fundoscopy and improvement in visual acuity bilaterally. Four months later, she had improved with +50% visual acuity in her left eye, and +60% visual acuity in the right eye.

## Discussion

### Approach to acute vision loss

**Figure 2.**
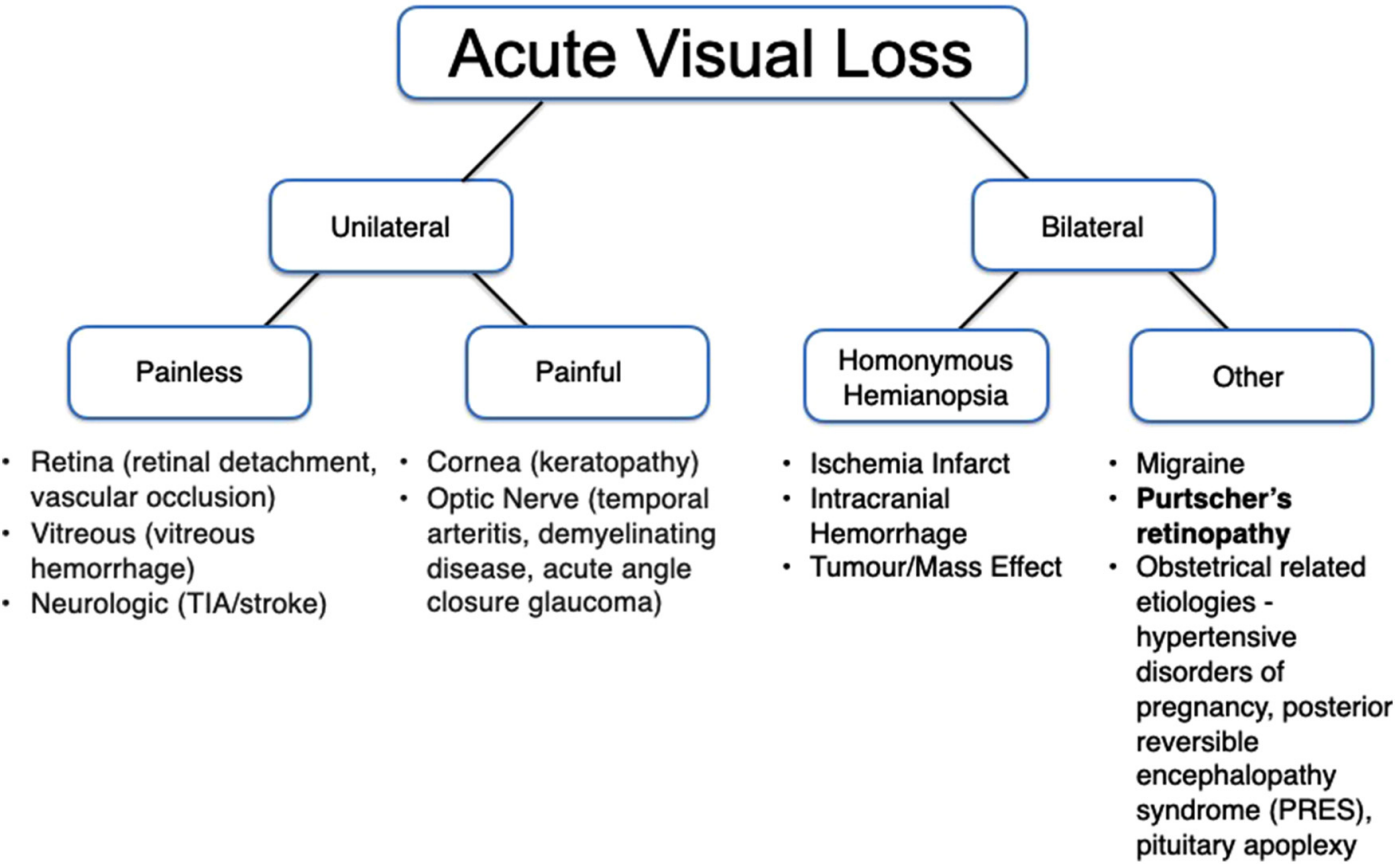
Approach to acute vision loss adapted from Bagheri and Mehta^1^ and Naderan^2^.

Acute persistent vision loss is an ophthalmologic emergency that necessitates urgent assessment. Etiologies of acute persistent vision loss can be organized according to unilateral or bilateral causes (Figure 2). In pregnancy, important causes such as hypertensive disorders of pregnancy, posterior reversible encephalopathy syndrome, hemolysis, elevated liver enzymes, low platelets (HELLP) syndrome or pituitary apoplexy should also be considered.^2^

## Purtscher's retinopathy

### Epidemiology

Purtscher's retinopathy was first described in 1910 by Otmar Purtscher in a patient with cranial trauma after falling from a tree.^3^ It is rare, affecting 2.4 cases per 10 million patients every year.^4^ It has a male predominance (60% male: 40% female) with a mean age of occurrence of 34 years.^5^ Purtscher's retinopathy is associated with trauma (33.8%), whereas Purtscher's-like retinopathy is the terminology used to describe systemic conditions including acute pancreatitis (19.1%), Valsalva maneuvre (8%), thrombotic thrombocytopenia purpura (7.3%), and pregnancy (4.4%).^4^

### Pathophysiology

The precise etiology of Purtscher's retinopathy remains elusive. Histopathologic studies suggest possible retinal vascular occlusion with edema of the retinal internal layers.^6^ Other theories propose microembolization, leading to arteriolar precapillary occlusion and microvascular infarcts of the retinal nerve fiber layers.^5^ Microembolization can occur from fat emboli in long bone fractures or from pancreatic proteases causing systemic damage in acute pancreatitis.^5^

Cases of Purtscher's-like retinopathy implicated in pregnancy are associated with gestational hypertension, HELLP syndrome, and postlabor.^6–9^ These are postulated to be due to complement-mediated leukocyte embolization in the context of compromised microvasculature, coupled with a hypercoagulable state of pregnancy.^6–9^

### Clinical signs

Clinical signs of Purtscher's retinopathy include loss of visual acuity and visual field defects.^4^ Retinal signs on fundoscopy include cotton wool spots (93%), retinal hemorrhages (65%), and Purtscher flecken (63%). Other signs include optic atrophy, mottling of retinal pigment epithelium, retinal thinning, and narrowing of retinal arteries.^5^

Interestingly, bilateral involvement tends to be associated with acute pancreatitis; whereas unilateral involvement is seen in trauma (e.g., chest compressions or long bone fractures).^4^

### Diagnosis

A diagnosis of Purtscher's retinopathy requires three of the following five criteria:

(1) presence of Purtscher flecken, (2) retinal hemorrhages, (3) cotton-wool spots, (4) probable or plausible etiology, and (5) investigations compatible with diagnosis (e.g., fluorescein angiography, ocular coherence tomography, visual field alterations, visual evoked potentials changes, or electroretinogram changes).^5^

### Management

Management of Purtscher's retinopathy is supportive and targeted toward correction of the underlying trigger. The use of systemic or local corticosteroids has been studied but is not correlated with improvements in visual acuity or time to recovery.^5^

Recovery is dependent on the etiology, severity of visual acuity deficits and fundoscopic abnormalities. Pancreatitis and trauma are associated with a higher probability of visual improvement.^5^ Poor prognostic factors include choroidal hypoperfusion, optic disc swelling, or massive deposition of Purtscher flecken.^10^

### Conclusions

Purtscher's retinopathy is a rare but important cause of acute vision loss associated with pancreatitis or maternal disease complicating pregnancy. Our case highlights Purtscher's-like retinopathy secondary to pancreatitis in pregnancy, and an approach for assessing acute vision loss in the pregnant patient.
